# Effect of Postrinsing Times and Methods on Surface Roughness, Hardness, and Polymerization of 3D-Printed Photopolymer Resin

**DOI:** 10.1055/s-0044-1786866

**Published:** 2024-05-17

**Authors:** Awutsadaporn Katheng, Wisarut Prawatvatchara, Watcharapong Tonprasong, Sahaprom Namano, Paweena Kongkon

**Affiliations:** 1 Department of Restorative Dentistry, Faculty of Dentistry, Naresuan University, Phitsanulok, Thailand; 2 Department of Prosthodontics, Faculty of Dentistry, Chulalongkorn University, Bangkok, Thailand; 3 Department of Gerodontology and Oral Rehabilitation, Graduate School of Medical and Dental Sciences, Tokyo Medical and Dental University, Tokyo, Japan

**Keywords:** degree of polymerization, postrinsing method, postrinsing time, stereolithography, surface hardness, surface roughness

## Abstract

**Objectives** This *in vitro* study investigated the effects of different postrinsing times and methods on the surface roughness, surface hardness, and degree of polymerization of materials manufactured via stereolithography (SLA).

**Materials and Methods** A total of 288 disk-shaped specimens were manufactured using an SLA three-dimensional (3D) printer. The specimens were randomly divided into nine groups ( *n* = 32) based on rinsing times and methods. The groups were categorized into three rinsing methods: automated, ultrasonic, and hand washing, with rinsing times of 5, 10, and 15 minutes using a 99% isopropanol alcohol as a solvent. Linear roughness (Ra) and area roughness (Sa) were measured using a 3D confocal laser microscopy; the roughness morphology was evaluated by using scanning electron microscopy. Vickers hardness (VHN) tests were performed using a Vickers microhardness tester. Fourier-transform infrared spectrometry was used to determine the degree of conversion of treated specimens.

**Statistical Analysis** Data were statistically analyzed using two-way analysis of variance. The post hoc Tukey tests were conducted to compare the differences between groups ( *p* < 0.05).

**Results** The choice of the rinsing time and method affected the surface properties of the SLA photopolymer resin. The 15 minutes of ultrasonic method exhibited the highest Ra scores (0.86 ± 0.1 µm), while the 15 minutes of automated method presented the highest Sa scores (1.77 ± 0.35 µm). For the VHN test, the 15 minutes of ultrasonic method displayed the highest VHN score (18.26 ± 1.03 kgf/mm ^2^ ). For the degree of polymerization, the 15 minutes of automated method was initially identified as the most effective (87.22 ± 6.80).

**Conclusion** To facilitate the overall surface roughness, surface hardness, and degree of polymerization, the optimal choice of postprocessing rinsing time and method for achieving a clear photopolymer resin was determined to be the ultrasonic method with a rinsing time of 15 minutes.

## Introduction

The implementation of computer-aided design and computer-aided manufacturing (CAD-CAM) systems has revolutionized the dental profession through the efficient production of a wide range of dental restorations, including occlusal splints, orthodontic appliances, fixed prostheses, and complete dentures. 1 In the realm of dental restoration manufacturing, two primary methodologies are used: subtractive methods and additive methods. Stereolithography (SLA) is an example of additive manufacturing (AM), which involves the gradual addition of materials, such as thermoplastic filaments or photocurable resins, to create the intended forms layer by layer. 2 SLA has notable advantages, including high printing resolution, smooth surface finish, intricate internal geometries, and minimal waste generation, rendering it a highly promising technology for various dental applications. 2 3 4

Three essential phases form the SLA process: data processing, manufacturing, and postprocessing. 5 6 Data processing involves utilizing CAM software to define printing and support parameters, as well as the slicing of data in the standard tessellation language (STL) format. The manufacturing step encompasses the additive fabrication of the interim restoration using a three-dimensional (3D) printer, while the final step involves postprocessing procedures, such as postrinsing and postpolymerizing. The objective of postrinsing is to remove any residual unpolymerized liquid resin from the printed object. Commonly used cleaning agents for postrinsing include isopropanol alcohol (IPA) and tripropylene glycol monomethyl ether (TPM). The surface characteristics, accuracy, mechanical characteristics, and cytotoxicity of AM dental resins can be radically affected by postrinsing. 3 7 8 Various parameters can be adjusted during the postrinsing process, such as the duration of rinsing, the cleaning method employed, and the choice of cleaning solution. 5 9

Surface properties play vital roles in determining the performance and longevity of dental prostheses. Surface roughness is particularly important because of its potential impact on the durability of dental prostheses. Increased roughness can result in minor tissue trauma, creating microorganism entrapping environments, and thereby contributing to tissue damage and oral diseases. 10 11 Surface hardness is an indicator of a material's capacity to withstand plastic deformation resulting from abrasive forces. The decreased hardness makes it prone to scratching and the formation of microcracks. Consequently, dental prostheses composed of polymethyl methacrylate may be compromised, creating favorable conditions for bacterial growth. The degree of polymerization plays a crucial role in determining the physical, chemical, and biological properties of denture base resins. 3 12 13 Elevated temperatures typically enhance the polymerization process. However, residual stress release, polymerization shrinkage, and thermal expansion can all contribute to the deformation of dental restorations overall. 14 Previous studies examining the impact of postrinsing time and methods on differences in polymerization have been inadequate. Therefore, further investigation is needed to understand how the postrinsing method and duration influences the final product.

Limited research has investigated potential variations in surface properties resulting from different postrinsing times and AM process methods. In their study, Ammoun et al 15 identified that hand washing with ultrasonics consistently produced better outcomes compared with automated methods. Regarding the impact of rinsing method and time on accuracy, Katheng et al 16 observed that the denture base resin was more precisely manufactured using the automated approach (rinsing times of 10 and 15 minutes) and the ultrasonic method (15 minutes). When examining the relationship between mechanical properties and rinsing method, “Yellow Magic” or centrifugal force revealed that specimens treated with IPA displayed reduced fracture loads in comparison to those who were washed. 17 For this reason, a variety of cleaning solutions are utilized, and extensive research has been conducted on the mechanical properties. 3 8 17 When comparing to various cleaning solution, it was reported that the specimen's trueness and precision increased with increasing concentration of TPM solvent after rinsing, in contrast to the IPA solvent group. 18 Additionally, with respect to the duration of rinsing, Lee et al 19 found that interim crowns that were rinsed with IPA for a period of 10 minutes had a high accuracy. The studies have indicated that SLA-printed orthodontic splint materials washed with IPA for a duration of less than 1 hour do not exhibit any obvious surface changes. 7

Despite manufacturer guidelines for specific materials and printers, the dental literature is lacking regarding the impact of rinsing times and methods on the surface roughness, surface hardness, and degree of polymerization. Consequently, the primary objective of this research endeavor was to examine the impact that different postrinsing times and methods have on the degree of polymerization, surface roughness, and surface hardness of photopolymer resins produced by SLA. The null hypothesis is assumed that there is no significant difference in the surface roughness, surface hardness, and degree of polymerization among different postrinsing times and methods.

## Materials and Methods

### Study Design and Workflow

**Fig. 1:**
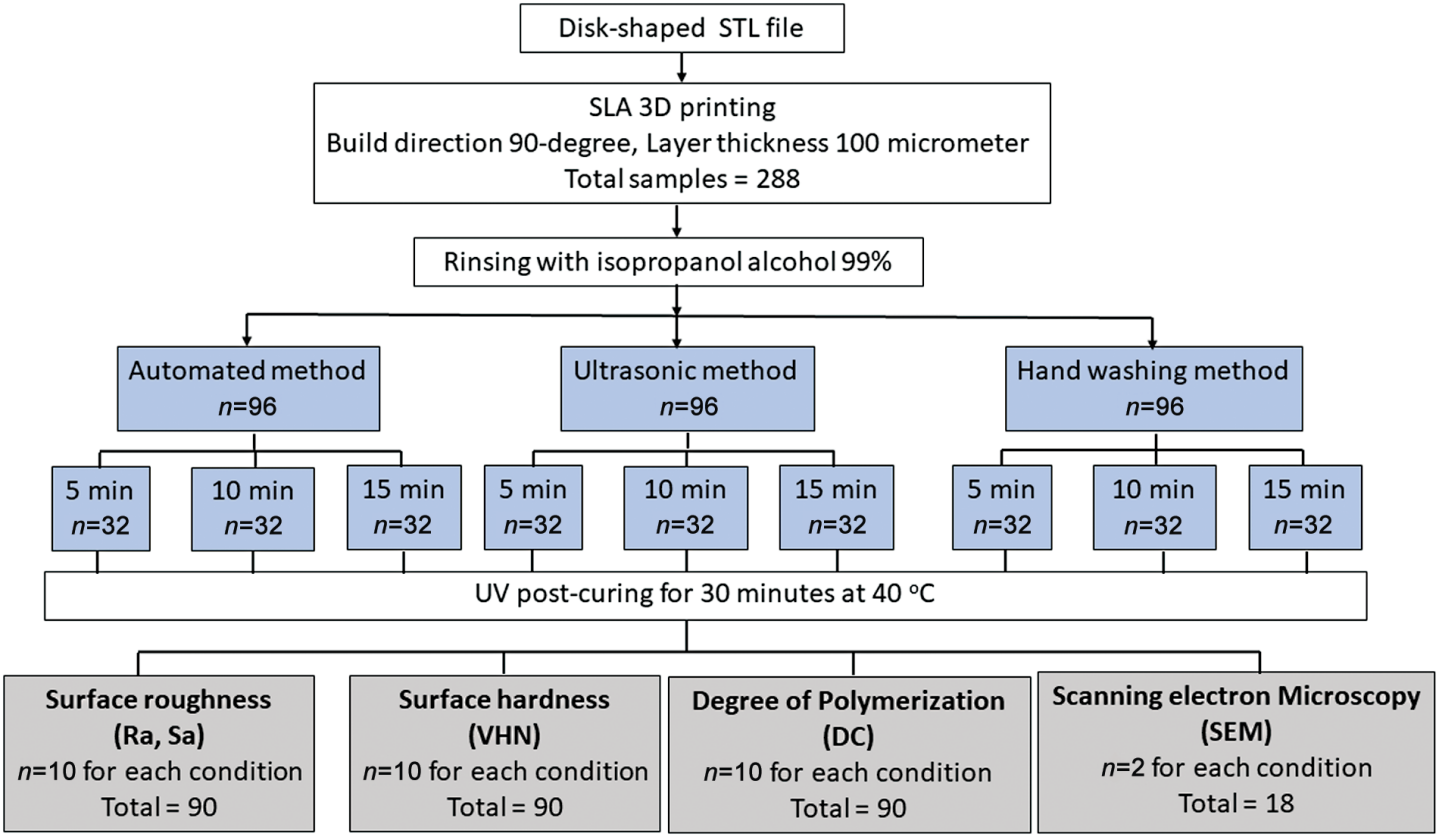
Flowchart of the experimental process for measuring the surface roughness, surface hardness, and degree of polymerization of specimens fabricated using stereolithography (SLA).

The study design and workflow of the experimental process are illustrated in Fig. 1 . A disk-shaped specimen that conforms to the International Organization for Standardization's recommended dimensions (ISO20795–1:2013), Dentistry—Base polymers, 20 was digitally designed using CAD software (Geomagic Freeform; 3D Systems, Rock Hill, South Carolina, United States). The specimen had a diameter of 10 mm and a height of 2 mm. The STL file of the specimen was imported into 3D printing software (PreForm software; Formlabs, Somerville, Massachusetts, United States). Support structures for the disc specimens were generated using slicing software. Two hundred eighty-eight specimens were manufactured using an SLA 3D printer (Form 2; Formlabs) with 90-degree angulation and 100-μm layer height, achieving a 25-μm *XY* resolution. The resin printing material utilized was a clear photopolymer resin (Formlabs). This resin is specifically designed for the tissue surfaces of hard splints and orthodontic appliances. The chemical composition of the resin is detailed in Table 1 . The printer was calibrated according to the manufacturer's recommendations including laser intensity, calibration between printer and software, and changes to resin properties for each individual batch, and all specimens were manufactured simultaneously. A prosthodontist (K.A.) performed all the procedures.

**Table 1:** Summary of 3D printing photopolymer resin used in this study

Product	Compositions	Manufacture
**Clear** resin	Methacrylate oligomer Methacrylate monomer Diphenyl (2,4,6-trimethyl benzoyl) phosphine oxide	Formlabs Inc., Somerville, Massachusetts, United States

Abbreviation: 3D, three-dimensional.

After printing, the specimens were meticulously detached from the built platform using a removal tool. Subsequently, 288 specimens were randomly divided into 9 groups ( *n* = 32) based on rinsing method (automated; A, ultrasonic; U, and hand washing; H) and three different rinsing times (5, 10, and 15 minutes), utilizing simple random sampling technique, aiming to achieve an unbiased representation of specimens in each group ( Fig. 1 ). Then, fresh 99% IPA solvent (KT Chemicals, Nishi, Osaka, Japan) was used to eliminate excess resin from all specimens.

### Automated Method

By means of an automated process, the specimens in the automated group were rinsed in accordance with the manufacturer's guidelines (Form Wash; Formlabs). During the specified rinsing times of 5, 10, and 15 minutes, the printed specimens remained attached to the constructed platform. The specimens were subsequently separated from the platform with care by employing a spatula.

### Ultrasonic Method

Before using the ultrasonic method, the specimens were rinsed with 99% in a wash bottle containing IPA for approximately 30 seconds. Following that, the specimens were submerged completely in a glass container containing IPA and subjected to the corresponding rinsing times in an ultrasonic bath (AU-16C Ultrasonic cleaner, Aiwa Medical Industry, Bunkyo, Tokyo, Japan).

### Hand Washing Method

**Fig. 2:**
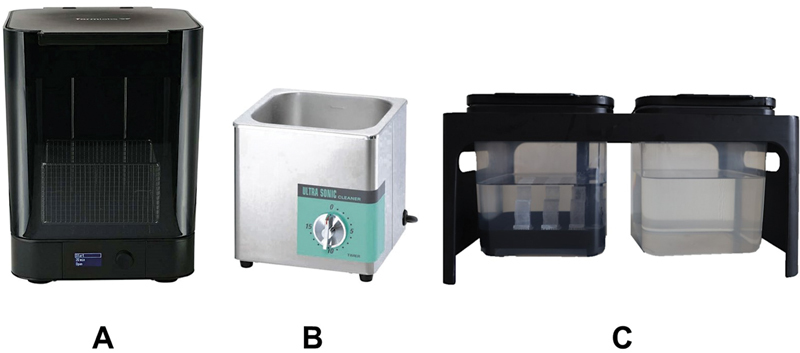
The three different equipment for rinsing methods: ( **A** ) automated method; ( **B** ) ultrasonic method; and ( **C** ) hand washing method.

The hand washing method utilized two plastic buckets with dimensions of 16 × 16 × 16 cm ^3^ from the Formlabs Finish Kit (Finish Kit, Formlabs) as shown in Fig. 2 . IPA solvent was added to the rinse bucket until it reached two-thirds of its volume. The specimens were placed in the rinse basket and were first rinsed by shaking the rinse basket for 30 seconds. Then, the basket and specimens were completely submerged in the solvent bath. The rinse basket and specimens were soaked for approximately half the total rinsing time. Thereafter, the rinse basket was shifted to the second rinse bucket and shaken for 30 seconds; subsequently, the basket and specimens were soaked for the remaining rinsing time.

Subsequently, all the specimens were carefully dried and placed in a ultraviolet (UV) polymerization machine (Form Cure, Formlabs) set at a temperature of 40°C for a duration of 30 minutes, as recommended in a previous study. 2 Following the removal of the support structures, the specimens were stored in a light-proof container at room temperature until the measurements were performed. All specimens from each group ( *n* = 32) were randomly divided into four different subgroups based on the experimental tests: the surface roughness ( *n* = 10), the surface hardness ( *n* = 10), the scanning electron microscopy (SEM) ( *n* = 2), and the degree of polymerization ( *n* = 10).

### Surface Roughness Assessment

The surface roughness assessment involved the measurement of linear roughness (Ra) and area roughness (Sa) in all specimens. Three random areas were observed along the linear midline traces located at the center of each specimen, with 2 mm between each observation point. These observations were recorded for 10 specimens, resulting in a total of 30 observations. Linear (transverse length: 2 mm) and area roughness was analyzed using a 3D confocal laser microscope (LEXT OLS4100; Olympus, Tokyo, Japan). A cutoff length of 80 μm was used for the analysis. Mean heights of the line and area roughness were recorded as Ra and Sa, respectively, in accordance with the guidelines outlined in ISO 25178–2:2012. 21 The average values of the traced areas were used for subsequent statistical analyses.

The surface morphology was investigated using a SEM. For each postrinsing method and different postrinsing times (5, 10, and 15 minutes), two samples were utilized. The surface of each sample was analyzed using a gold spotter-coated sample (gold sputtering unit, JEOL Ltd., Akishima, Japan), and the observations were made at a 1,000× magnification using the SEM (JSM-IT500HR, JEOL Ltd., Tokyo, USA).

### Vickers Hardness Test

Vickers hardness tests were performed using a microhardness tester (Zhu-S, Indentec, West Midlands, United Kingdom). An indentation with a load of 300 g was applied at the midpoint of the base material for a dwell time of 15 second using a diamond tip. Each specimen was measured three times on one side at the center point with 2 mm between measurements. This resulted in 10 specimens and 30 observations. The pyramids created using the square-based pyramid indenter were evaluated. The diagonals of the square-shaped traces were measured using a stereomicroscope (SZX16, Olympus) to determine the Vickers hardness number (VHN). The average values of the three VHN tests were calculated and recorded. VHN was calculated using the following equation:



where VHN denotes the Vickers hardness, *F* denotes the applied load (kgf), and *d* ^2^ is the indentation area in square millimeters (mm ^2^ ).

### Degree of Conversion

Fourier-transform infrared spectroscopy analysis was performed in attenuated total reflectance mode (ATR, Nicolet iS50; Thermo Fisher Scientific). This analysis was performed to identify the functional groups present in the 3D-printed resin and to assess the degree of polymerization. Disc-shaped specimens were positioned at the center of the ATR crystal to ensure optimal contact ( *n* = 10). The experiment was repeated three times for each group. The degree of conversion (DC) was determined by analyzing the changes in the peak height ratios of the absorbance (Abs) intensities between specific functional groups in the 3D-printed specimens. An aliphatic C = C peak at 1638 cm ^−1^ and the C–H reference peak at 777 cm ^−1^ were used for this purpose. DC was calculated using the following equation:



where *Abs*_1638_ is the absorbance intensity of the aliphatic C = C peak and *Abs*_reference_ is the absorbance intensity of the C–H reference peak.

### Statistical Analysis

Statistical analysis was conducted using a statistical software program (IBM SPSS Statistics, v24.0; IBM Corp., Armonk, New York, United States). The normality of data distribution was assessed using the Shapiro–Wilk test, and the homogeneity of variances was evaluated using the Levene test. To analyze the effects of the postrinsing method and time on surface roughness, surface hardness, and degree of polymerization, a two-way analysis of variance (ANOVA) was performed. Post hoc Tukey tests were conducted to compare differences between groups. The significance level ( *α* ) for all statistical tests was set at 0.05. Based on previous literature, 16 the sample size calculation for this study used an analytical software program (G*Power 3.1.9.2; Kiel University, Kiel, Schleswig-Holstein, Germany). A sample size of 10 per condition was required to obtain an effect size *f* of 0.45 and 80% power at 5% *α* error.

## Results

**Fig. 3:**
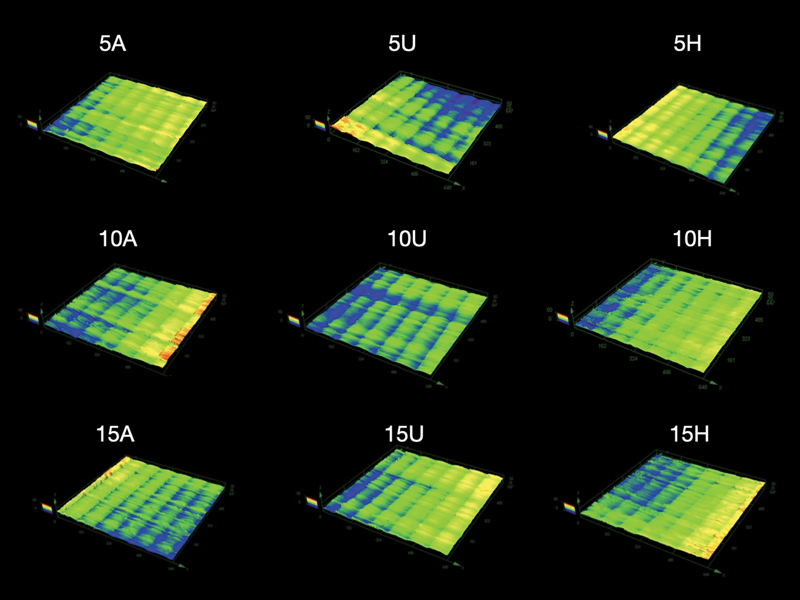
Representative images of the specimens captured using a three-dimensional (3D) confocal laser microscope. Numbers 5, 10, and 15 denote rinsing times of 5, 10, and 15 minutes, respectively. The letters A, U, and H represent automated, ultrasonic, and hand washing, respectively.

**Fig. 4:**
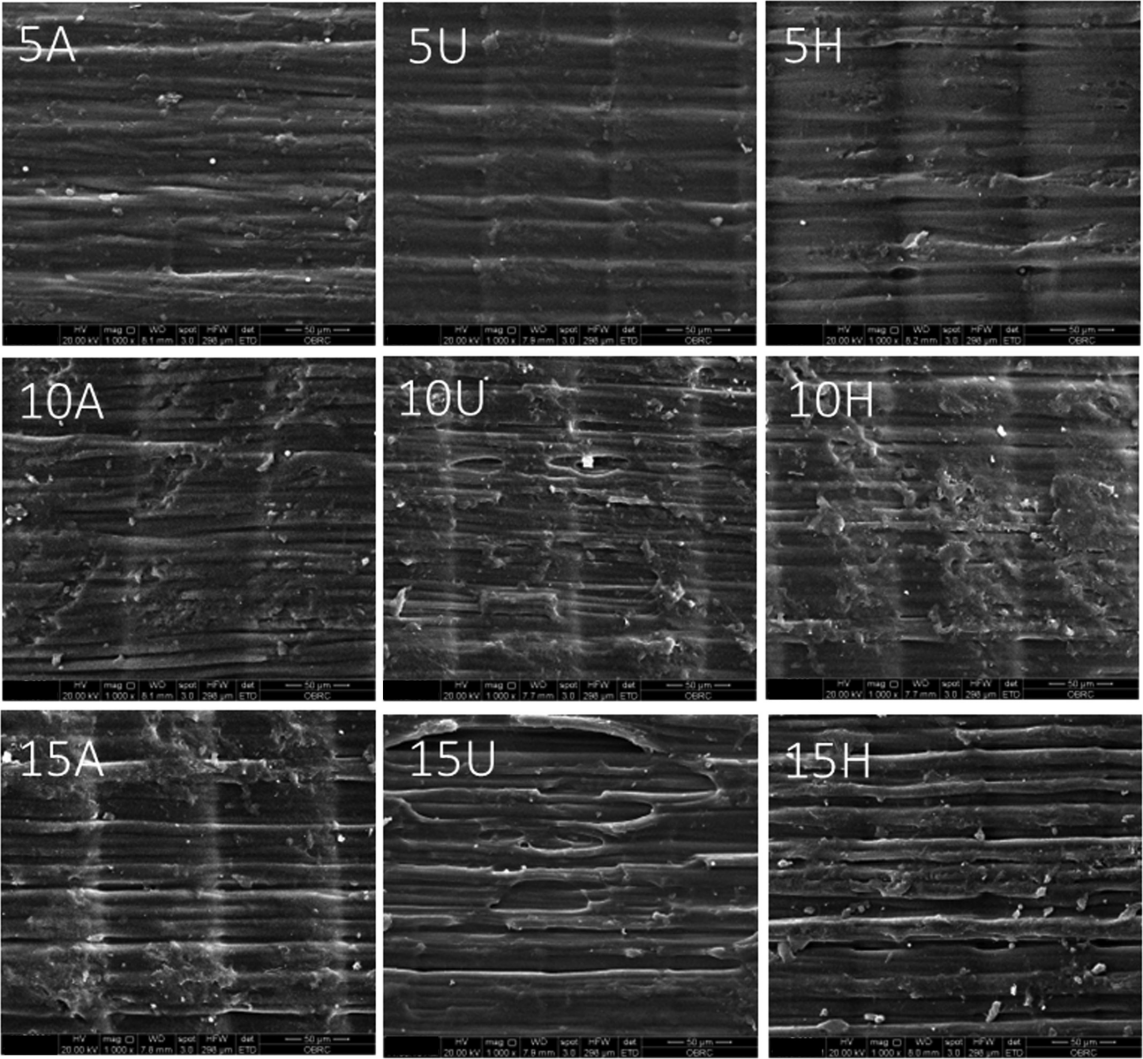
Surface morphology of the specimens by using the scanning electron microscope (SEM) at 1,000× magnification. Numbers 5, 10, and 15 denote rinsing times of 5, 10, and 15 minutes, respectively. The letters A, U, and H represent automated, ultrasonic, and hand washing, respectively.

The choice of the rinsing time and method affected the surface properties of the SLA photopolymer resin. Table 2 summarizes the results of the two-way ANOVA. Representative images of the specimens captured using a 3D confocal laser microscope are shown in Fig. 3 . The SEM results are shown in Fig. 4 .

**Table 2:** Summary of ANOVA results for the surface roughness (Ra, Sa), surface hardness (VHN), and degree of polymerization (DC) of 3D-printed photopolymer resin specimen under different postrinsing methods and various time conditions

Test	Effect	df	*F*	*p*
**Ra**	Methods	2	15.803	< 0.001 a
	Times	2	11.661	< 0.001 a
	Methods × Times	4	10.374	< 0.001 a
**Sa**	Methods	2	11.456	< 0.001 a
	Times	2	0.568	0.569
	Methods × Times	4	2.318	0.064
**VHN**	Methods	2	187.82	< 0.001 a
	Times	2	62.01	< 0.001 a
	Methods × Times	4	56.46	< 0.001 a
**DC**	Methods	2	1.195	0.308
	Times	2	3.605	0.032 a
	Methods × Times	4	8.830	< 0.001 a

Abbreviations: 3D, three-dimensional; ANOVA, analysis of variance; DC, degree of conversion; Ra, linear roughness; Sa, area roughness; VHN, Vickers hardness number.

Note: df represents the numerator's degree of freedom.

a Indicates significant differences between the groups.

The mean and standard deviation of the surface roughness values is listed in Tables 3 and 4 . Statistical analysis using two-way ANOVA revealed that both the postrinsing method and time had a significant impact on Ra ( *F* = 15.803 and 11.661, respectively, *p* < 0.001). Furthermore, a significant interaction was observed between methods and time ( *F* = 10.374, *p* < 0.001). The post hoc multiple comparison test indicated significant differences ( *p* < 0.05) between groups. The Ra scores ranged from 0.69 ± 0.08 to 0.86 ± 0.10 μm. The 15U group exhibited the highest Ra score, while the 5H group obtained the lowest Ra score which did not significantly differ from the 5U, 10H, and 5A groups.

**Table 3:** Arithmetic mean height of the lines (Ra) of 3D-printed photopolymer resin specimen under three different postrinsing methods and various time conditions

Rinsing method	Ra (µm, *n* = 10/group)					
	Rinsing time					
	5 min		10 min		15 min	
	Mean	SD	Mean	SD	Mean	SD
Automated	0.76 ^cdef^	0.10	0.79 ^abcd^	0.08	0.80 ^abc^	0.09
Ultrasonic	0.71 ^ef^	0.12	0.85 ^ab^	0.08	0.86 ^a^	0.10
Hand washing	0.69 ^f^	0.08	0.72 ^def^	0.07	0.78 ^bcde^	0.06

Abbreviations: 3D, three-dimensional; Ra, linear roughness; SD, standard deviation.

Note: Different superscript letters indicate statistically significant differences ( *p* < 0.05).

**Table 4:** Arithmetic mean height of the surface (Sa) of 3D-printed photopolymer resin specimen under three different postrinsing methods and various time conditions

Rinsing method	Sa (µm, *n* = 10/group)					
	Rinsing time					
	5 min		10 min		15 min	
	Mean	SD	Mean	SD	Mean	SD
Automated	1.56 ^abc^	0.38	1.69 ^ab^	0.34	1.77 ^a^	0.35
Ultrasonic	1.19 ^c^	0.22	1.33 ^abc^	0.20	1.44 ^abc^	0.18
Hand washing	1.29 ^bc^	0.51	1.24 ^c^	0.16	1.54 ^abc^	0.26

Abbreviations: 3D, three-dimensional; Sa, area roughness; SD, standard deviation.

Note: Different superscript letters indicate statistically significant differences ( *p* < 0.05).

Significant differences were observed in mean Sa scores among the three postrinsing methods ( *F* = 11.456, *p* < 0.001). However, no significant interaction was found between the methods and time points ( *F* = 2.318, *p* = 0.064), and time did not significantly affect mean Sa scores ( *F* = 0.568, *p* = 0.569). The post hoc multiple comparison test indicated significant differences ( *p* < 0.05) between groups. As shown in Table 4 , the Sa scores ranged from 1.19 ± 0.22 to 1.77 ± 0.35 μm, where the 15A group had the highest Sa score. While the 5U group had the lowest Sa score; however, it was not significantly different from the 5H, 10H, 15H, 5U, 10U, 15U, and 5A groups. The confocal images as shown in Fig. 3 reveal that the sample surface of 5 minutes postrinsing time exhibited partial smoothness, indicating the presence of residual uncured resin on the surfaces. In contrast, the surfaces displayed irregular fissured surfaces, likely resulting from the layering process during printing. Although the quantitative color values representing specific height or slope may exhibit slight variations between groups, the Ra and Sa scores can be employed to analyze the surface topography effectively. The SEM results are represented in Fig. 4 . It was observed that sediment accumulation persisted on the surface of the specimens in the groups that underwent a postrinsing process of 5 and 10 minutes. This accumulation led to the surface exhibiting some degree of light reflectivity. In contrast, specimens exposed to a 15-minute postrinsing time exhibited more exposed printed layer surfaces compared with the other groups.

The results of the Vickers hardness test presented in Table 5 show that the VHNs ranged from 11.93 ± 0.70 to 18.26 ± 1.03 kgf/mm ^2^ . Two-way ANOVA revealed that both the postrinsing method and time had a significant effect on surface hardness ( *F* = 187.82 and 62.01, respectively, *p* < 0.001). Furthermore, there was a significant interaction between the method and time ( *F* = 56.46, *p* < 0.001). The post hoc multiple comparison test indicated significant differences ( *p* < 0.05) between groups. The 15U group showed the highest surface hardness, followed by the 10U, 15H, and 10H groups in that order. Nevertheless, no significant difference was observed between the 15H and 10U groups.

**Table 5:** Vickers hardness (VHN) of 3D-printed photopolymer resin specimen under three different postrinsing methods and various time conditions

Rinsing method	Surface hardness (kgf/mm ^2^ , *n* = 10/group)					
	Rinsing time						
	5 min		10 min		15 min	
	Mean	SD	Mean	SD	Mean	SD
Automated	11.93 ^f^	0.70	12.53 ^f^	0.64	13.66 ^de^	1.23
Ultrasonic	13.93 ^cd^	0.88	16.26 ^b^	0.70	18.26 ^a^	1.03
Hand washing	12.73 ^ef^	0.70	14.73 ^c^	0.70	16.06 ^b^	0.79

Abbreviations: 3D, three-dimensional; SD, standard deviation; VHN, Vickers hardness number.

Note: Different superscript letters indicate statistically significant differences ( *p* < 0.05).

For the degree of polymerization (DC), the results of the two-way ANOVA showed no significant differences between the postrinsing methods ( *F* = 1.195, *p* = 0.308), but there was a significant difference between postrinsing times ( *F* = 3.605, *p* = 0.032). Furthermore, a significant interaction was observed between the time and method ( *F* = 8.830, *p* < 0.001). The post hoc multiple comparison test indicated significant differences ( *p* < 0.05) between groups. The DC scores ranged from 73.41 to 87.22% ( Table 6 ). The 15A group had the highest DC score (87.22 ± 6.80) but it was not significantly different from the 15H, 15U, and 10H groups, while the lowest DC score was observed in the 5A group.

**Table 6:** Degree of polymerization (DC) of 3D-printed photopolymer resin specimen under three different postrinsing methods and various time conditions

Rinsing method	Degree of polymerization (%, *n* = 10/group)					
	Rinsing time						
	5 min		10 min		15 min	
	Mean	SD	Mean	SD	Mean	SD
Automated	73.41 ^c^	3.25	77.60 ^bc^	8.62	87.22 ^a^	6.80
Ultrasonic	76.83 ^bc^	2.66	78.59 ^bc^	1.35	80.22 ^abc^	7.45
Hand washing	78.12 ^bc^	3.22	80.99 ^ab^	4.11	82.79 ^ab^	4.96

Abbreviations: 3D, three-dimensional; DC, degree of conversion; SD, standard deviation.

Note: Different superscript letters indicate statistically significant differences ( *p* < 0.05).

## Discussion

3D-printed photopolymer resin, similar to light-cured resin composite materials, utilizes the oxygen-sensitive free radical polymerization mechanism. The detrimental impact of oxygen on photopolymerization leads to the formation of a sticky layer consisting of uncured dimethacrylate monomer, hence compromising the surface properties. Although the leftover layer is considered unappealing, the oxygen-inhibited partial curing of the surface during 3D printing methods is advantageous for interfacial bonding of layer-by-layer structures during printing processes. The purpose of postrinsing is to eliminate this layer prior to the postpolymerization process. The residual oxygen-inhibited polymerization layer cannot undergo UV curing during the postpolymerization process due to the lack of free radicals, which in this study influenced the surface roughness, surface hardness, and degree of polymerization.

Based on the results of this study, postrinsing time and method influenced the surface roughness, surface hardness, and degree of polymerization in at least one group; therefore, the null hypothesis was rejected. However, the difference in efficacy could be attributed to the measurement methods used. 22 Generally, the rinsing time depends on the 3D-printed material. Additionally, factors such as resin and printer type, 23 printer manufacturer's guidelines, 24 25 and specific postprocessing requirements can also influence the results. 26

Regarding linear roughness (Ra), a longer rinsing time resulted in an increase in linear roughness. The results showed that after 15 minutes of postrinsing, the ultrasonic method had the highest mean Ra score (0.86 ± 0.01 μm) but was not significantly different from the automated method (0.80 ± 0.09 μm), while hand washing (0.78 ± 0.06 μm) had significantly lower value than both the ultrasonic and automated methods.

Postrinsing refers to the step in which a printed object is submerged in a solvent, typically IPA, to remove excess or uncured resin from its surface. The duration of the postrinsing step can affect the final surface finish of a 3D-printed object. 27 Insufficient postrinsing time may result in the presence of residual uncured resin, or other contaminants, on the surface of the printed object, resulting in a rougher finish, as it can solidify and create irregularities or small bumps. However, longer postrinsing times facilitate more thorough removal of uncured resin from the printed object's surface. The findings of the current investigation indicate that the Ra score was highest in the 15-minute postrinsing time group for all rinsing methods. As compared with the 10- and 5-minute groups, which correspond to the SEM result, the less sediment accumulation and more exposed printing layer surface are related to the high Ra score in the 15-minute postrinsing time group. However, previous studies have shown that extended postrinsing times can negatively affect the surface topography of 3D-printed resins, leading to surface fissures when the rinsing time is increased to 12 hours. This was likely due to solvent molecules diffusing into the polymer network, causing surface swelling. 7 28

There was a significant difference in mean Sa scores between the three methods. Multiple comparison analyses showed that at 15 minutes of rinsing time, the ultrasonic method had the lowest Sa score (1.44 ± 0.18 μm), followed by the hand washing (1.54 ± 0.26 μm) and automated methods (1.77 ± 0.35 μm). However, there was no significant difference in the mean scores between the hand washing and ultrasonic methods. The method used for postrinsing can also affect surface roughness. In contrast, previous studies have found no statistically significant difference in surface roughness when comparing the rinsing times of 1 to 10 minutes for automated method. 29 The variability in findings across studies may be attributed to differences in the compositions of printing resin materials and printing protocols utilized in these studies. 7 29

Different postrinsing methods had varying effects on the final surface texture. Immersion and agitation are two common methods for postrinsing. The immersion method is relatively gentle and suitable for removing excess resin; however, it may not be as effective in eliminating fine details or achieving a highly polished surface finish. Agitation techniques, such as mechanical agitation or ultrasonic cleaning, involve actively moving or agitating the printed object in a rinsing solvent. 15 16 30 Agitation can enhance resin removal by dislodging any remaining resin particles and improving the overall cleanliness of the object's surface. 30 According to the findings of this study, hand washing may be comparable to immersion, whereas ultrasonic cleaning, which resembles agitation, produces smoother surface finishes.

Regarding the degree of polymerization, the two-way ANOVA indicated no significant differences between the methods, but a significant difference between the times. An insufficient postrinsing time may result in the presence of residual uncured resin or contaminants on the printed object's surface. These residues can interfere with the degree of polymerization and the process. 31 32 The degree of polymerization may be influenced by the efficacy of the postrinsing procedure, which ensures the elimination of uncured resin and reduces the existence of unreacted monomers. 33 The findings indicated that automated procedures rinsing for 15 minutes produced the greatest DC score; however, no statistically significant differences were seen among the other methods. When postrinsing time is taken into account, a longer postrinsing time may facilitate the removal of residues from the uncured resin layer, therefore increasing the surface object's exposure to UV radiation and enabling polymerization to occur during the postpolymerizing stage. This promotes further polymerization and cross-linking, leading to a higher degree of polymerization and improved mechanical properties.

Microhardness is a crucial indicator of surface structural stability. In addition, this property may indicate scratching or brushing resistance in dental restorations. Previous studies have indicated that microhardness is influenced by the chemical composition and denture prefabrication techniques. 34 35 36 Focusing on methacrylate-based polymers, the most used dental appliance, molecular crosslinks can be formed by polymerization. However, incomplete polymerization can occur, resulting in residual monomer retention, which leads to increased water absorption and decreased mechanical properties. 28 In this study, the duration and rinsing technique had a significant impact on the hardness value of a 3D-printed denture base. The amount of residual monomer can be decreased by extending the washing protocol, as described in a previous study. 28 In addition, a positive correlation between surface hardness and DC has been reported. 37 Although the postrinsing time of automated method for clear photopolymer resin, as per recommendation from the manufacturer, is 10 minutes, the results of this study show that the automated method at 15-minute rinsing times had higher surface hardness (13.66 ± 1.23 kgf/mm ^2^ ) than 10 minutes (12.53 ± 0.64 kgf/mm ^2^ ). Furthermore, when compared with different rinsing methods, for 15-minute rinse durations, the ultrasonic approach achieves the highest in surface hardness (18.26 ± 1.03 kgf/mm ^2^ ). It is possible that the agitation from ultrasonication might be the most effective rinsing process for removing uncured resin compared with other techniques. In addition, the higher the rinsing time interval, the higher the hardness level detected.

The clinically recommended postprocessing approach involves using the ultrasonic method with a 15-minute rinsing time, optimizing overall surface roughness, material hardness, and degree of polymerization. This guidance is essential for dental professionals, aiding them in selecting postrinsing protocols that enhance biocompatibility, material durability, and structural integrity in 3D-printed dental devices. Despite the higher Ra score associated with the ultrasonic method with a 15-minute rinsing time, its effectiveness in achieving improved hardness and polymerization outweighs the potential drawback of increased roughness. The study underscores the intricate interplay between postrinsing parameters and their collective impact on the final characteristics of 3D-printed photopolymer resin, emphasizing the need for balanced considerations in dental applications and promoting standardized postprocessing procedures for consistent outcomes.

Limitations of this study include its *in vitro* design, excluding variations in intraoral conditions. It is important to consider that these study results are not directly comparable to all 3D-printed resins currently available in the market. Therefore, the results may vary if the parameters are changed during 3D printing and postcuring. Achieving an optimal surface finish depends on factors other than postrinsing, such as printing technology, resin properties, curing process, and subsequent postprocessing steps. Furthermore, specific recommendations for the postrinsing time and method may vary depending on the printer manufacturer, resin supplier, and the desired surface quality for a particular application.

## Conclusion

Based on the findings of this *in vitro* study, we concluded that rinsing time and method influenced the surface roughness, surface hardness, and degree of polymerization. To facilitate the overall surface roughness, surface hardness, and degree of polymerization, the ultrasonic method with a rinsing time of 15 minutes is optimal choice for achieving a clear photopolymer resin.
